# Determinants of multimorbidity in low‐ and middle‐income countries: A systematic review of longitudinal studies and discovery of evidence gaps

**DOI:** 10.1111/obr.13661

**Published:** 2023-12-17

**Authors:** Michelle M. C. Tan, Matheus G. Barbosa, Pedro J. M. R. Pinho, Esubalew Assefa, Ana Á. M. Keinert, Charlotte Hanlon, Barbara Barrett, Alexandru Dregan, Tin Tin Su, Devi Mohan, Cleusa Ferri, Graciela Muniz‐Terrera, Matthew Prina, MUTUAL consortium

**Affiliations:** ^1^ Department of Health Service and Population Research, Institute of Psychiatry, Psychology & Neuroscience King's College London London UK; ^2^ Global Public Health, Jeffrey Cheah School of Medicine and Health Sciences Monash University Malaysia Sunway City Selangor Malaysia; ^3^ South East Asia Community Observatory (SEACO) Monash University Malaysia Sunway City Selangor Malaysia; ^4^ Victorian Heart Institute Faculty of Medicine, Nursing and Health Sciences, Monash University, Clayton Campus Clayton Victoria Australia; ^5^ Psychogeriatric Unit, Department of Psychiatry, Medical School Universidade Federal de São Paulo (UNIFESP) São Paulo Brazil; ^6^ Centre for Innovative Drug Development and Therapeutic Trials for Africa (CDT‐Africa) Addis Ababa University Addis Ababa Ethiopia; ^7^ Department of Economics, College of Business and Economics Jimma University Jimma Ethiopia; ^8^ Department of Economics, Faculty of Arts and Social Sciences The Open University Milton Keynes UK; ^9^ Global Mental Health, Centre for Global Mental Health, Institute of Psychiatry, Psychology and Neurosciences King's College London London UK; ^10^ Department of Psychiatry, School of Medicine, College of Health Sciences Addis Ababa University Addis Ababa Ethiopia; ^11^ Edinburgh Dementia Prevention University of Edinburgh and Western General Hospital Edinburgh UK; ^12^ Department of Social Medicine, Heritage College of Osteopathic Medicine Ohio University Athens Ohio USA

**Keywords:** determinants, longitudinal studies, low‐ and middle‐income countries, multimorbidity

## Abstract

Multimorbidity—the coexistence of at least two chronic health conditions within the same individual—is an important global health challenge. In high‐income countries (HICs), multimorbidity is dominated by non‐communicable diseases (NCDs); whereas, the situation may be different in low‐ and middle‐income countries (LMICs), where chronic communicable diseases remain prominent. The aim of this systematic review was to identify determinants (including risk and protective factors) and potential mechanisms underlying multimorbidity from published longitudinal studies across diverse population‐based or community‐dwelling populations in LMICs. We systematically searched three electronic databases (Medline, Embase, and Global Health) using pre‐defined search terms and selection criteria, complemented by hand‐searching. All titles, abstracts, and full texts were independently screened by two reviewers from a pool of four researchers. Data extraction and reporting were according to Preferred Reporting Items for Systematic Reviews and Meta‐Analyses (PRISMA) guidelines. Methodological quality and risk of bias assessment was performed using the Newcastle‐Ottawa Scale for cohort studies. Data were summarized using narrative synthesis. The search yielded 1782 records. Of the 52 full‐text articles included for review, 8 longitudinal population‐based studies were included for final data synthesis. Almost all studies were conducted in Asia, with only one from South America and none from Africa. All studies were published in the last decade, with half published in the year 2021. The definitions used for multimorbidity were heterogeneous, including 3–16 chronic conditions per study. The leading chronic conditions were heart disease, stroke, and diabetes, and there was a lack of consideration of mental health conditions (MHCs), infectious diseases, and undernutrition. Prospectively evaluated determinants included socio‐economic status, markers of social inequities, childhood adversity, lifestyle behaviors, obesity, dyslipidemia, and disability. This review revealed a paucity of evidence from LMICs and a geographical bias in the distribution of multimorbidity research. Longitudinal research into epidemiological aspects of multimorbidity is warranted to build up scientific evidence in regions beyond Asia. Such evidence can provide a detailed picture of disease development, with important implications for community, clinical, and interventions in LMICs. The heterogeneity in study designs, exposures, outcomes, and statistical methods observed in the present review calls for greater methodological standardisation while conducting epidemiological studies on multimorbidity. The limited evidence for MHCs, infectious diseases, and undernutrition as components of multimorbidity calls for a more comprehensive definition of multimorbidity globally.

**Abbreviations**
ADL: activities of daily living
BMI: body mass index
CHARLS: China Health and Retirement Longitudinal Study
CI: confidence interval
CKB: China Kadoorie Biobank
COPD: chronic obstructive pulmonary disease
HDL‐C: high‐density lipoprotein cholesterol
HICs: high‐income countries
HIV: human immunodeficiency virus
HR: hazard ratio
IADL: instrumental activities of daily living
JIN: Jiangsu Nutrition Study
LMICs: low‐ and middle‐income countries
mg/dL: milligrams per deciliter
MHCs: mental health conditions
MI: myocardial infarction
MUTUAL: investigating MUltimorbidity ThroUgh cApacity buiLding
NCDs: non‐communicable diseases
NOS: Newcastle‐Ottawa Scale
OR: odds ratio
PRISMA: Preferred Reporting Items for Systematic Reviews and Meta‐Analyses
RR: relative risk
SES: socio‐economic status
SHARE: Survey of Health, Ageing and Retirement in Europe
TUA: Towards Useful Aging
vs: versus
WC: waist circumference
WHT.5R: waist divided by height^0.5^
WHtR: waist‐to‐height ratio

## 1 INTRODUCTION

Multimorbidity, commonly defined as the co‐occurrence of two or more chronic health conditions in an individual,^1^ represents a considerable immediate and future challenge for health and social care systems around the world. Management of multimorbidity has been typically configured around the single disease model rather than taking an integrated and person‐centered approach, often resulting in fragmented healthcare and conflicting medical advice. The global prevalence of multimorbidity has been increasing throughout the 21st century as a consequence of increased life expectancy, population aging, and lifestyle risk factors; low‐ and middle‐income countries (LMICs) are no exception to this increasing burden.^2^, ^3^, ^4^ It is estimated that approximately one in three individuals across the globe lives with multimorbidity.^3^ The implications for individuals and societies are enormous, whereby multimorbidity leads to poorer quality of life,^5^ greater functional decline,^6^ higher mortality rates,^7^ and increased healthcare^8^ and social care expenditures.^9^ The Academy of Medical Sciences UK has positioned multimorbidity as a priority for global health research,^4^ placing it at the forefront of research and healthcare.

In response to this challenge, there has been an explosion of research investigating multimorbidity. The vast majority of multimorbidity studies have been cross‐sectional in design, from high‐income countries (HICs), and focused on older adults. These findings, however, cannot be generalized to LMICs and populations with other age groups. Although snapshot analyses are useful for understanding the prevalence of multimorbidity and the clustering of diseases, they provide little information on the development of multimorbidity over time and the sequencing of the onset of different health conditions. Longitudinal studies are required to estimate the risk, understand the onset of multimorbidity, and identify potential causal pathways.^4^ Although there has been an increased focus on longitudinal approaches worldwide, there are few systematic reviews of longitudinal multimorbidity in LMICs. A recent scoping review of longitudinal trajectories of multimorbidity predominantly focused on HICs from the Global North (34 studies from HICs and 1 from an upper‐middle‐income country).^10^ In that review, multimorbidity onset and progression were driven by older age, higher socio‐economic and area‐level deprivation, being overweight, and poorer health behaviors. Two recent systematic reviews focused on the prevalence of multimorbidity,^11^ or analsed a single risk factor for multimorbidity development among subjects with overweight or obesity.^12^ Other reviews generally focused on scoping older cross‐sectional studies in one region (i.e., South Asia),^13^ or solely considered non‐communicable diseases (NCDs).^2^, ^14^ To our knowledge, none have focused on longitudinal investigations of determinants of multimorbidity consisting of both communicable and NCDs among the general populations in LMICs. There is an urgent need to establish what is currently known about prospective determinants of multimorbidity observed within LMICs.

In parallel with NCDs, the emergence of COVID‐19, leading to enduring health conditions (e.g., long COVID), generated extra pressure on already overwhelmed, unprepared, and under‐sourced health systems. These developments emphasize the need to understand the epidemiology of multimorbidity in LMICs to inform the development of context‐specific guidelines and interventions for managing multiple chronic conditions in resource‐constrained health systems. Consequently, this could ensure better health service provision, health management, and resource deployment aligning the increasing number of people living with multimorbidity. Therefore, the aim of this review was to evaluate long‐term risk and protective factors associated with multimorbidity in tandem with their potential underlying mechanisms by synthesizing existing evidence from prospective longitudinal studies in LMICs.

## 2 METHODS

### 2.1 Search strategy

This review follows the Preferred Reporting Items for Systematic Reviews and Meta‐Analyses (PRISMA) 2020 statement and checklist.^15^ See the checklist in Supplementary Table S1. The study protocol was registered with PROSPERO (Registration code: CRD42021275137). A comprehensive online literature search using the Ovid interface, of three electronic databases, Medline, Embase, and Global Health, was systematically carried out, including studies from inception to August 2021. We used a pre‐defined structured search strategy combining linguistic variations of text words as well as Medical Subject Headings (MeSH) terms for multimorbidity (e.g., “multimorbidity”, “multi‐morbidity”, “multi morbidity”, “multiple chronic conditions”, “multiple comorbidity”, and “multiple chronic disease”) and LMICs. These were combined by the Boolean operators “AND” and “OR”. Although “comorbidity” is now known to be distinct from “multimorbidity”, the terms have been used synonymously in the past. Therefore, we included “comorbidity” in the search algorithm, and studies of multiple comorbidities with no specific index disease were considered eligible. LMICs were as defined by the Cochrane Filters 2020 based on the World Bank Country and Lending Groups 2019 list of LMICs (https://epoc.cochrane.org/lmic-filters). The final search terms are presented in Supplementary Table S2. No limits were placed on the language or the year of publications. Backward and forward citations were also hand‐searched for any related articles not identified in the database search, with a final update in August 2022. These references were included in the pool of references to be screened and were subjected to the same screening processes as those retrieved from databases.

### 2.2 Eligibility criteria

#### 2.2.1 Inclusion criteria

Peer‐reviewed longitudinal studies focused on multimorbidity in population‐ or community‐based settings in LMICs were considered for inclusion. For inclusion, studies needed to contain an explicit list of chronic conditions (which included NCDs, mental health conditions [MHCs], and chronic communicable diseases) to account for multimorbidity and outcomes of multimorbidity that included determinants or its synonyms (e.g., risk factors, predictors, or factors associated with). No restrictions were applied on the type and number of chronic diseases. Chronic conditions could be assessed through self‐reported clinical diagnosis, clinical assessment, medication use, medical record data, and/or laboratory investigations.

#### 2.2.2 Exclusion criteria

- Studies that selected populations based on the presence of a pre‐defined index disease.
- Studies that involved only persons living in institutions or recruited from secondary and tertiary hospitals, hospices, rehabilitation centers, nursing homes, or prisons. We aimed to understand multimorbidity in the general population and not in a selected group.
- Qualitative studies, cross‐sectional studies, and questionnaire validation studies.
- Articles not reporting full original research, including systematic reviews, review articles, literature reviews, case reports, opinion pieces, conference proceedings, editorials, letters, newsletters, commentaries, debates, policy reviews, books, dissertations, and abstracts.

### 2.3 Data extraction

The Bibliography Management Programme EndNote 20 (Thomson Reuters, New York, USA) was used to create and manage the search library. All duplicate references were removed using the Ovid database deduplication function, and the potentially eligible studies were all imported to EndNote. Duplicates not detected by this function were removed manually during the subsequent screening. The references were also uploaded to Rayyan, an intelligent web‐tool for managing systematic reviews.

To minimize bias, a two‐stage screening process was employed to select relevant studies from those identified in our search. Firstly, initial screening of all titles and abstracts was undertaken independently by four reviewers in pairs (MGB, PJMRP, EA, and AÁMK) in Rayyan to identify studies that met the inclusion criteria. In the second stage, full texts of the potentially eligible studies were retrieved and assessed for eligibility. Each pair of reveiwers independently screened half subset of the titles and abstracts and full texts of the potential studies. Once all reviewers had completed this task, their results were discussed to resolve discrepancies. Disagreements were mediated by MMCT and MP and resolved through consensus.

**FIGURE 1:**
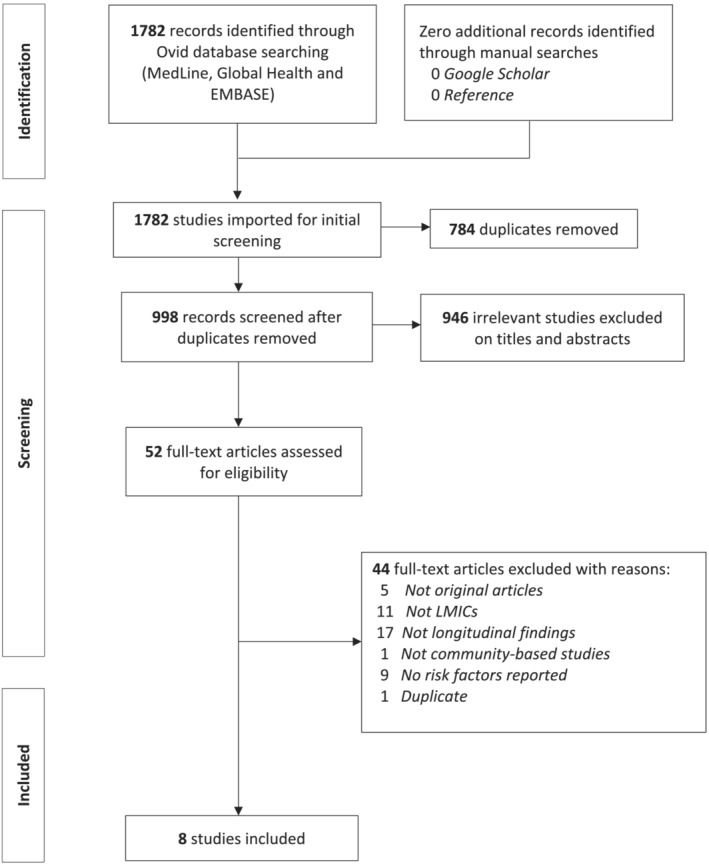
Preferred Reporting Items for Systematic Reviews and Meta‐Analyses (PRISMA) flowchart demonstrating the process for study selection.

Information from the selected articles was extracted by three authors (MGB, PJMRP, and AÁMK) using a standardized proforma developed for this systematic review. MMCT verified the extracted data and retrieved further information needed from the articles. The proforma/data extraction form was prepared and piloted by MMCT and finalized by discussion with the senior authors (GMT and MP) (see the data extraction form in Supplementary Tables S3). For each article, the following details were extracted: authors; publication year; country; study data (year, data collection period, source, recruitment settings, sample size); demographic data (age average and range, gender); definition of multimorbidity; measures of medical diagnosis; type and number of chronic conditions considered in the studies; prevalence, incidence, or change in multimorbidity over time; determinants of multimorbidity and their details such as effect estimate of association; statistical methods adopted; and significance values. The corresponding author of an included article where relevant data could not be retrieved was contacted by email to request the information required for inclusion. Figure 1 displays the number of articles retained at each point of the review procedure and the reasons for exclusion.

### 2.4 Risk of bias assessment

The Newcastle‐Ottawa Scale (NOS) for cohort studies^16^ was used to assess the methodological quality and risk of bias of each study. This was independently carried out by two assessors (MMCT and EA). Any discordance arising in the quality assessment of studies was addressed by consensus and in consultation with the senior authors (GMT and MP). The NOS consists of eight items, classified into three domains of potential bias, namely, “Selection”, “Comparability”, and “Outcome”. A study can be given a maximum of one star for each item within the Selection and Outcome domains, whereas a maximum of two stars can be given for the Comparability domain.

### 2.5 Data synthesis

Although this review included studies that applied simple counts of chronic diseases to assess multimorbidity, these studies were too heterogeneous for a meta‐analysis in terms of population characteristics, data source, types of effect size estimate, analytical strategies (statistical analyses and adjustments for different confounders/covariates), and the number of conditions included in the multimorbidity calculation. Therefore, included studies were narratively synthesized to describe the focus of this review, informed by Popay and colleagues' guidelines.^17^

## 3 RESULTS

### 3.1 Literature search

Using the search strategy as described, our search yielded 1782 studies. After the removal of 784 duplicates, we screened the titles and abstracts of 998 studies. We excluded 946 records on titles and abstracts that were not longitudinal multimorbidity studies in LMICs and were not original articles. The full texts of 52 studies were assessed for inclusion using the review criteria specified in the methods section. Forty‐four studies were further excluded after the full‐text screening because they were not longitudinal studies (in terms of study design and analysis) (*n* = 17), they were conducted in HICs (*n* = 11), they did not assess risk factors or determinants of multimorbidity (*n* = 9), they were not original articles (*n* = 5), they were not community‐ or population‐based (*n* = 1), and they were duplicates (*n* = 1). A study conducted in Chile^18^ (data collection period 1990–2020) was included in the present review, despite Chile becoming a HIC in 2012, given the baseline was collected while it was a LMIC, and that Chile is an under‐served population with large within‐country inequalities, portraying a similar profile to LMICs. Therefore, eight studies were included in the systematic review for final data synthesis (Figure 1), all published in the last decade, with half published in the year 2021.

### 3.2 Study characteristics

Details of the characteristics of the included studies are summarized in Table 1. Most studies were conducted in upper‐middle‐income countries and in Asia. Five studies were conducted in China,^19^, ^20^, ^21^, ^22^, ^23^ and one each in Armenia,^24^ Malaysia,^25^ and Chile,^18^ with studies starting between 1990 and 2020. The follow‐up periods ranged between 1.5 and 30 years. One study reported results from multiple countries of various economic status (i.e., China vs. 28 HICs and Bulgaria from the SHARE [Survey of Health, Ageing and Retirement in Europe] panel database in a composite),^22^ of which the relevant China findings were included in the current review. Out of 5 studies conducted in China, 3 primarily derived data from the China Health and Retirement Longitudinal Study (CHARLS), with different chronic conditions considered for varied outcomes of multimorbidity. The remaining two Chinese studies used data from the Jiangsu Nutrition Study (JIN) and the China Kadoorie Biobank (CKB), respectively.

**TABLE 1:** Characteristics of included studies (*n* = 8)^a^.

Author, year	Country	Data collection period	Sample size	Age (years)	Sex (% female)	Data source/settings	Measures of multimorbidity and list of chronic conditions	Number of diseases	Prevalence/Incidence of multimorbidity
Demirchyan et al. (2013)	Armenia	1990–2012	725	39–90	81.5	Cohort of survivors of the 1988 Earthquake in north Armenia, urban representative	Self‐reported physician‐diagnosed chronic diseases Hypertension MI Heart diseases (excluding MI) Diabetes Stroke Asthma Respiratory diseases (excluding asthma) Migraine Allergic diseases (excluding asthma) Gastrointestinal diseases Arthritis/Joint pain Back pain Kidney/Urinating problems Cancer Mental/Psychological problems Disabilities	16	Prevalence Baseline: Total sample: 76.7% Older age group (≥65 years old): 86.7% Younger age group (39–64 years old): 72.5%
Ruel et al. (2014)	China	2002–2007	1020	20–60+	58.0	Jiangsu longitudinal Nutrition Study (JIN)	Blood test Anemia Blood test and self‐reported use of medication Diabetes Hypercholesterolemia Blood pressure measurement and self‐reported use of medication Hypertension Self‐reported physician‐diagnosed chronic diseases Arthritis Hepatitis Coronary heart disease Asthma Stroke Fracture Cancer	11	Prevalence Baseline: 14% Follow‐up: 34%
Hussin et al. (2019)	Malaysia	2014–2016	729	60+	49.8	Towards Useful Aging (TUA) study	Self‐reported physician‐diagnosed acute and chronic diseases Hypertension Hyperlipidemia Diabetes Stroke Osteoarthritis Heart diseases Cataract/glaucoma Renal failure Asthma COPD Tuberculosis Gout Hip fracture Thyroid disorders Cancer	15	Incidence Follow‐up: 18.8% with no disease at baseline developed multimorbidity (incidence rate: 13.7/100 person‐years); 40.9% who had one disease at baseline developed multimorbidity (incidence rate: 34.2/100 person‐years)
Blümel et al. (2020)	Chile	1990–2020	1,066	40–59	100.0	*Servicio de Salud Metropolitano Sur* (*Hospital Barros Luco*, Santiago de Chile), Chile. All women who attended the service for preventive healthcare check‐ups were invited, which is mandatory for public servants on an annual basis.	SIGGES (*Sistema de Gestión de Garantías Explícitas de Salud*) Hypertension Arthrosis (hip or knee) Diabetes Depression Cancer (breast, ovary, uterine cervix or colorectal) Acute MI Stroke COPD Chronic kidney disease Parkinson's disease HIV	11	Prevalence Baseline: 4.6% Follow‐up: 49.7%
Yang et al. (2021)	China	2011–2015	14,093	45–101	52.1	China Health and Retirement Longitudinal Study (CHARLS)	Self‐reported physician‐diagnosed chronic diseases Hypertension Diabetes Cancer Lung disease Heart problems Stroke Psychiatric problems Arthritis Dyslipidemia Liver disease Kidney disease Digestive disease Asthma Memory problems	14	Prevalence^b^ Baseline: Men: 37.5% Women: 42.7%
Han et al. (2021)	China	2004–2014	461,047	30–79	59.0	China Kadoorie Biobank (CKB)	Medical records Ischemic heart disease Stroke Type 2 diabetes	3	Incidence^c^ Follow‐up: 3.1%
Qiao et al. (2021)	China	2011–2015	7,883	45+	48.9	CHARLS	Self‐reported physician‐diagnosed chronic diseases Hypertension Diabetes Cancer or malignant tumor Chronic lung disease Heart problem Stroke Emotional/Psychiatric disease Stomach or other digestive disease Arthritis/Rheumatism Kidney disease Liver disease Memory‐related disease Asthma	13	Incidence Follow‐up: 45.8% participants handicapped in ADL (vs. control group: 27.3%); 38.6% in IADL (vs. control group: 27.4%) developed multimorbidity
Lu et al. (2021)	China	2011–2015	10,521	45+	Cardiometabolic multimorbidity: 58.9 No cardiometabolic multimorbidity: 51.5	CHARLS	Self‐reported physician‐diagnosed chronic diseases Diabetes Stroke Heart problems	3	Incidence Follow‐up: 1.3% with no baseline disease developed cardiometabolic multimorbidity; 12.4% with one baseline disease developed cardiometabolic multimorbidity

Abbreviations: ADL, activities of daily living; COPD, chronic obstructive pulmonary disease; HIV, human immunodeficiency virus; IADL, instrumental activities of daily living; MI, myocardial infarction; vs., versus.

^a^ All studies defined multimorbidity as the presence of two or more diseases within a single individual.

^b^ Prevalences of multimorbidity were computed by summation of the percentages of two, three, four, and five or more chronic diseases reported in the original studies.

^c^ Incidence of multimorbidity = (number of participants who developed cardiometabolic multimorbidity reported in the original studies/total number of participants who were free of cardiometabolic disease in the original studies) × 100% = 14,164/461,047 × 100% = 3.1%.

**TABLE 2:** Protective and risk factors associated with multimorbidity reported by the included studies.

Author, year	Adjusted protective and risk factors^a^
Demirchyan et al. (2013)	**Protective factors** Current good social support (OR: 0.4, 95% CI: 0.28–0.70) and current university/higher education (OR: 0.56, 95% CI: 0.36–0.87). **Risk factors** Number of stressful life events (OR: 1.15, 95% CI: 1.07–1.25), current low affordability of healthcare (OR: 2.36, 95% CI: 1.56–3.58), perceived poor living standards during post‐earthquake of 10 years (OR: 1.68, 95% CI: 1.10–2.57), and baseline BMI (OR: 1.06, 95% CI: 1.01–1.11). The findings were further confirmed by the calculation of adjusted relative risks: **Risk factors** Baseline BMI (RR: 1.01, 95% CI: 1.00–1.02), perceived poor living standards during the post‐earthquake decade (RR: 1.12, 95% CI: 1.03–1.22), and the number of stressful life events (RR: 1.03, 95% CI: 1.02–1.04).
Ruel et al. (2014)	**Protective factors** A high consumption of fruits and vegetables (*p* < 0.05), and grain products (other than rice and wheat) (*p* < 0.001) were associated with healthier stages in the evolution of multimorbidity.
Hussin et al. (2019)	**Risk factors** Subjects without any disease at baseline: Women (adjusted OR: 3.378, 95% CI: 1.391–8.197), smoking (adjusted OR: 3.260, 95% CI: 1.079–5.177), and irregular involvement in food preparation (a component of leisure physical activities) (adjusted OR: 2.363, 95% CI: 1.061–3.365) were significantly related to multimorbidity incidence at follow‐up. Subjects with one disease at baseline: BMI 22–27 kg/m^2^ (adjusted OR: 2.240, 95% CI: 1.142–4.395) and inadequate daily intake of iron (adjusted OR: 0.921, 95% CI: 0.857–0.990) were significantly related to multimorbidity incidence at follow‐up.
Blümel et al. (2020)	**Risk factors** Obesity (adjusted OR: 2.48, 95% CI: 1.71–3.61), having a job that did not require a qualification (and lacking higher education) (adjusted OR: 2.18, 95% CI: 1.67–2.83) and low HDL‐C (≤50 mg/dL) (adjusted OR: 1.31, 95% CI: 1.02–1.68).
Yang et al. (2021)	**Protective factors** Childhood food adequacy (reflecting childhood severe deprivation) was associated with a lower number of chronic diseases at baseline (men: −0.171, 95% CI: −0.245 to −0.097; women: −0.223, 95% CI: −0.294 to −0.152) and a slower rate of change in multimorbidity (men: −0.015 per year, 95% CI: −0.027 to −0.003; women: −0.012 per year, 95% CI: −0.024 to −0.001). **Risk factors** Parental physical abuse was associated with increased number of chronic diseases (intercept: 0.119, 95% CI: 0.033–0.205 for men and 0.268, 95% CI: 0.188–0.348 for women) and a higher rate of increase (slope: 0.013, 95% CI: 0.000–0.027 for men and 0.022, 95% CI: 0.008–0.036 for women) in multimorbidity.
Han et al (2021)	**Risk factors** Individual lifestyle factors and HR for conversion from first cardiometabolic disease to cardiometabolic multimorbidity were tobacco smoking (HR: 1.07, 95% CI: 1.02–1.12), excessive alcohol drinking (HR: 1.07, 95% CI: 1.01–1.13), less healthy dietary habits (HR: 1.04, 95% CI: 0.94–1.16), low physical activity (HR: 1.08, 95% CI: 1.05–1.12), and unhealthy body shape (HR: 1.27, 95% CI: 1.23–1.31).
Qiao et al (2021)	**Risk factors** Participants with functional limitations (assessed by either ADL or IADL) had an increased risk of developing multiple long‐term chronic conditions compared with their respective controls. The adjusted ORs of developing chronic illnesses in the following groups were: ADL disability group: 1.68 (95% CI: 1.31–2.16) for developing 1 disease, 2.58 (95% CI: 1.97–3.38) for developing 2 diseases, 3.22 (95% CI: 2.31–4.49) for developing 3 diseases, and 4.10 (95% CI: 2.58–6.51) for developing ≥4 diseases; IADL disability group: 1.16 (95% CI: 0.96–1.39) for developing 1 disease, 1.46 (95% CI: 1.19–1.80) for developing 2 diseases, 1.79 (95% CI: 1.36–2.37) for developing 3 diseases, and 2.55 (95% CI: 1.69–3.84) for developing ≥4 diseases.
Lu et al. (2021)	**Risk factors** Obesity indicators: From no baseline disease to having cardiometabolic multimorbidity: WHtR (OR: 1.76, 95% CI: 1.05–2.97), WC (OR: 2.06, 95% CI: 1.29–3.27), WHT.5R (OR: 1.81, 95% CI: 1.16–2.83), BMI (OR: 1.48, 95% CI: 0.98–2.24); From one baseline disease to cardiometabolic multimorbidity: WHtR (OR: 2.04, 95% CI: 1.24–3.35), WC (OR: 1.89, 95% CI: 1.29–2.77), WHT.5R (OR: 1.86, 95% CI: 1.24–2.78), BMI (OR: 1.47, 95% CI: 1.06–2.04).

Abbreviations: ADL, activities of daily living; BMI, body mass index; CI, confidence interval; HDL‐C, high‐density lipoprotein cholesterol; HR, hazard ratio; IADL, instrumental activities of daily living; mg/dL, milligrams per deciliter; OR, odds ratio; RR, relative risk; WC, waist circumference; WHtR, waist‐to‐height ratio; WHT.5R, waist divided by height^0.5^.

^a^ Except studies by Blümel et al.^18^ where crude ORs were reported.

The total number of participants across the eligible studies was 497,084, with sample sizes ranging from 725 to 461,047. The age of adults at baseline varied between 20 and 101 years old. All studies involved both men and women, except for the study from Chile. The number of health conditions included in the studies ranged from 3 to 16, with all studies including diabetes, heart disease, and stroke as part of the multimorbidity measure. All the studies adopted a disease count method as a measure of multimorbidity, with self‐reported physician‐diagnosed chronic diseases being the most common type of measure.

All studies reported using the most common definition of multimorbidity—the co‐occurrence of two or more chronic diseases within a single individual (Table 1). The only difference was observed in Lu and colleagues' studies,^23^ where only three cardiometabolic diseases (i.e., diabetes, stroke, and heart disease) were considered in the measure of multimorbidity. Hence, multimorbidity was termed “cardiometabolic multimorbidity” across their article. Of 4 studies that reported incidence of multimorbidity at follow‐up, the rates ranged from 1.3% to 40.9%.^21^, ^22^, ^23^, ^25^ In comparison with participants who were free from any disease at baseline, those with one disease at baseline were found to have higher rates of developing multimorbidity at follow‐up.^23^, ^25^ Among 3 studies that explored the overall baseline prevalence of multimorbidity, the prevalence varied from 4.6% to 76.7%^18^, ^19^, ^24^ (Table 1). In the studies conducted in Armenia,^24^ the older age group (≥65 years old) had a higher prevalence of multimorbidity than the younger age group (39–64 years old) (86.7% vs. 72.5%). Of 2 studies that investigated baseline prevalence of multimorbidity with follow‐up prevalence reported, an upward trend of multimorbidity was observed in follow‐up from baseline (baseline: 14%, 5‐year follow‐up: 34%,^19^ and baseline: 4.6%, 30‐year follow‐up: 49.7%^18^).

### 3.3 Protective and risk factors for multimorbidity

Table 2 summarizes the determinants of multimorbidity reported by the selected longitudinal studies.

#### 3.3.1 Socio‐economic factors, markers of social inequities, and childhood adversity

Three studies examined a range of socio‐economic determinants and markers of social inequities on multimorbidity. The findings from a multi‐ethnic Malaysian cohort confirmed associations found in cross‐sectional analyses, with women having higher levels of multimorbidity in LMICs.^25^ Results of the longitudinal study conducted in Armenia^24^ indicated that low socio‐economic status (SES) and social inequities were indeed associated with incident multimorbidity. Perceived low affordability of healthcare services, poor living standards, and lower education level were independent predictors of incident multimorbidity.^24^ Additionally, stressful life events and poor social support were among the psychosocial determinants responsible for the incident multimorbidity ascertained from the Armenian earthquake survivors. One study that included results for Chinese males and females aged 45–101 years showed that parental physical abuse during childhood was associated with an increased number of chronic diseases and an increase in the risk of multimorbidity in adulthood.^20^ This study also determined that adequate food intake during childhood (reflecting the severity of childhood deprivation) was a protective factor against the number of chronic diseases at baseline and the rate of change in multimorbidity over the 4 years of follow‐up. The study conducted among women in Chile^18^ indicated that individuals with unqualified jobs (in general with lower education) had a 40% higher odds of developing multimorbidity at 30 years of follow‐up.

#### 3.3.2 Lifestyle behaviors

Three studies investigated lifestyle behaviors,^19^, ^21^, ^25^ with healthy behaviors shown to be substantially associated with reduced severity of multimorbidity. Greater consumption of fruits, vegetables, and grain products (other than rice and wheat) was associated with healthier stages in the evolution of multimorbidity in a Chinese cohort.^19^ In a cohort of Malaysian elderly aged 60 years and above, inadequate daily iron intake (assessed using a 7‐day dietary history questionnaire) was significantly associated with higher multimorbidity incidence at 1.5 years of follow‐up among participants with one disease at baseline.^25^ Among participants without any disease at baseline in Malaysia, smoking and irregular involvement in food preparation (considered by the authors as a component of leisure physical activities) were significantly related to multimorbidity incidence at follow‐up.^25^ It is important to note that irregular involvement in food preparation may also imply dining out frequently and consuming fast foods or processed foods among the households. A study among 461,047 adults in China reported similar results in lifestyle factors where the following increased the hazard ratio for conversion from first cardiometabolic disease to cardiometabolic multimorbidity: tobacco smoking, excessive alcohol drinking, less healthy dietary habits, low physical activity, and unhealthy body shape.^21^

#### 3.3.3 Obesity

Four studies found that having obesity was associated with developing or worsening multimorbidity. Demirchyan et al.^24^ reported that each one‐point increase in BMI (kg/m^2^) increased the chance of developing incident multimorbidity by 6% over 22 years of observation in the Armenian cohort. A study focused on the interplay between cardiometabolic multimorbidity and obesity (as measured by BMI, waist‐to‐height ratio, waist circumference, and waist divided by height^0.5^) found that all the increasing obesity indicators studied were significantly associated with cardiometabolic multimorbidity.^23^ This was the first longitudinal study to investigate the association of baseline obesity indicators with the risk of developing cardiometabolic multimorbidity among participants free of baseline cardiometabolic multimorbidity. Blümel and coauthors^18^ found that obesity (defined by BMI of ≥30 kg/m^2^) was the strongest risk factor for multimorbidity in older women in Chile. A study conducted in Malaysia observed a BMI range of 22–27 kg/m^2^ was associated with an increased risk of multimorbidity incidence at follow‐up.^25^ Conversely, the study sample was older population and Asians, and the BMI cut‐off values used for obesity were not specified. Hence, results shall be interpreted with caution, particularly because the BMI cut‐off for obesity category for Asian populations is always lower compared with the WHO classification.^26^

#### 3.3.4 Disability

One study that explored the association between functioning and multimorbidity showed that people with lower levels of functioning had an increased risk of developing chronic conditions in comparison with their control groups.^22^ Functioning was assessed by the participants' reporting of any difficulty in the activities of daily living (ADL)^27^ and the instrumental activities of daily living (IADL) scales,^28^ respectively.

#### 3.3.5 Dyslipidemia

Dyslipidemia at baseline, characterized by a low high‐density lipoprotein cholesterol (HDL‐C) in a homogeneous cohort of Chilean women, was associated with 1.31‐fold higher multimorbidity during follow‐up.^18^

### 3.4 Risk of bias assessment

Detailed results of the studies' risk of bias assessment based on the NOS system for cohort studies are depicted in Table 3. Overall, only two studies were allocated full stars across the three NOS domains. All the 8 studies were allocated a star (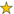) for representativeness of exposed cohort, selection of the non‐exposed cohort for drawing the exposed cohort from the same community, and demonstration that outcome of interest was not present at the start of the study all under the Selection domain. Almost all the studies were rated a star for ascertainment of exposure from secured records and/or structured interviews (Selection domain). Five out of 8 studies were allocated 2 stars for comparability of cohorts based on their design or statistical analysis being controlled for confounders, 2 studies were given 1 star each under this item, and 1 publication received no star for this, in which a maximum of 2 stars can be rated under the Comparability domain. Under the Outcome domain, half of the studies were rated a star for assessing outcomes based on an independent blind assessment or a record linkage. All the studies were deemed to have an acceptable length of follow‐up period for outcomes to occur, defined by 6 months or longer. Only half of the 8 studies were rated a star for adequacy of follow‐up of cohorts, with complete follow‐up or <20% loss to follow‐up (Outcome domain).

**TABLE 3:** Summary of the review authors' judgments for each risk of bias domain and item.

Author, year	Selection				Comparability	Outcome		
	Representativeness of the exposed cohort (maximum 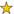 )	Selection of the non‐exposed cohort (maximum 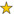 )	Ascertainment of exposure (maximum 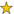 )	Demonstration that outcome of interest was not present at start of study (maximum 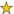 )	Comparability of cohorts on the basis of the design or analysis (maximum 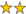 )	Assessment of outcome (maximum 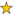 )	Was follow‐up long enough for outcomes to occur (maximum 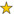 )	Adequacy of follow‐up of cohorts (maximum 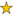 )
Demirchyan et al. (2013)^24^	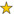	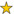	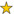	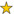	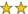	◯	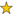	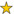
Ruel et al. (2014)^19^	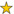	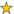	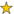	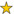	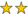	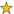	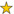	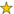
Hussin et al. (2019)^25^	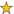	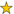	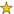	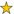	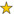	◯	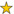	◯
Blümel et al. (2020)^18^	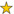	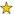	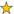	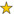	◯	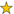	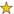	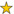
Yang et al. (2021)^20^	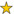	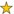	◯	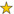	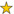	◯	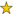	◯
Han et al. (2021)^21^	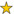	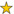	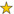	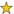	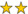	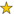	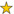	◯
Qiao et al. (2021)^22^	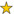	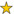	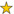	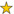	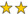	◯	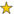	◯
Lu et al. (2021)^23^	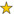	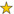	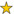	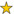	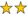	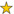	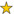	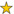

## 4 DISCUSSION

The development of healthcare delivery models that adequately respond to the complex situation of multimorbidity in LMICs requires clarity on the epidemiology within these contexts. In this review, we assessed determinants of multimorbidity in LMICs. We identified eight eligible studies, confined to three countries in Asia and one in South America. Despite the growing prevalence of chronic diseases, the evidence base for multimorbidity and its determinants is extremely limited and not evenly distributed among LMICs. All studies were published within the past 10 years, with half published in 2021. The leading chronic conditions reported in the included studies in this review were heart disease, stroke, and diabetes; and there was a lack of consideration of mental health problems, communicable diseases, and undernutrition in the existing evidence.

Limited resources for research, including access to funding for longitudinal studies and infrastructure to diagnose multiple health conditions in LMIC settings, could be possible reasons for this paucity. However, this is a significant research gap, particularly considering the high burden of multimorbidity. The lag in studying multimorbidity may have also contributed to the delay in designing effective interventions for those living with multimorbidity. In the long term, a better understanding of multimorbidity will contribute towards the development of evidence‐based and context‐specific health and social care policies, service planning, and delivery programs, building on the ongoing shift from specialized to integrated healthcare systems and from disease‐centered to person‐centered health and rehabilitative care for people living with multimorbidity. Based on the available recent evidence synthesized in this review, particular determinants appear to play crucial roles in the development of multimorbidity in these settings. We will now consider these determinants in detail.

### 4.1 Determinants of multimorbidity

#### 4.1.1 Socio‐economic factors, markers of social inequities, and childhood adversity

Our review indicated that social adversity and inequity factors are distinctively affecting the development of multimorbidity in LMICs. For instance, the determinants of incident multimorbidity identified in the Armenian population^24^ indicated that low SES and inequities pose a serious threat to both individual and public health‐related outcomes. Educational level was shown to be a significant determinant of multimorbidity and is a well‐established marker of social inequities in health studies, including the included studies conducted in Chile.^18^, ^29^, ^30^ The effect of higher education on a lower risk of multimorbidity in the Chilean population was postulated to be due to higher SES, healthier behaviors, and perceived better knowledge in individuals with higher education compared with those with lower education. The authors in Armenia described that those who reported poor affordability of healthcare were at a higher risk of having incident multimorbidity. This finding was consistent with an earlier study also conducted in Armenia, which identified low healthcare affordability as an independent predictor of self‐rated poor health among women.^31^ When considering the higher need for utilization of healthcare services among those suffering from multimorbidity, the low affordability of these essential services becomes an urgent and critical issue to address. In this sample, those who reported lower living standards during the 10 years following the earthquake had higher odds of suffering from multimorbidity in the later stage of life, suggesting material conditions were among the factors explaining the role of social inequalities in multimorbidity. It is interesting to note that the current objective SES score (ranging from 0 to 24) measured in the study was not associated with incident multimorbidity in the final modeling.^24^ This association was instead largely mediated by the perceived living standards during the decade following the earthquake, indicating a lag time for detrimental effects of poor socio‐economic conditions and their impact on multimorbidity outcomes.

Increasing numbers of stressful life events were associated with a higher risk of incident multimorbidity. The risk of developing multimorbidity increased independently by 3% for each additional stressful life event over a participant's lifetime, suggesting that experiencing stressful events during the lifespan might be a more important factor for incident multimorbidity than age itself. Nevertheless, it is worth noting that the stressful life events experienced by the participants in the included study were more severe than those usually observed in the general population. This was exacerbated by extremely high rates of poverty (i.e., >45% of the population) in the earthquake zone, as the study population was a cohort of earthquake survivors who lived in extremely difficult conditions during the last two decades. Therefore, caution is needed when generalizing the findings of this study to other populations. Good social support, however, was found to reduce the chance of incident multimorbidity over twofold. The positive impact of adequate social support on a population's health status, including multimorbidity, has been highlighted in various other studies,^32^, ^33^, ^34^ suggesting the importance of building well‐functioning social networks in reducing multimorbidity. It could be the unmeasured contribution of mental health that is important here, or it could be about information sharing, being nudged to seek help or access to care. Strategies targeting the reduction of such inequities, along with strengthening social networks, may considerably reduce multimorbidity.

Childhood adversity, specifically parental physical abuse and food deprivation during childhood, was associated with multimorbidity in later life in a longitudinal and large nationally representative cohort in China.^20^ It was proposed that childhood adversity could affect the future SES and shape unhealthy lifestyle practices in adulthood,^35^, ^36^ although there is little direct evidence in this study to support this hypothesis. The findings, on the one hand, imply that the risk of multimorbidity related to childhood adversity accumulates over the life course. On the other end of the spectrum, it shows that the dramatic economic development in China and the marked increase in living standards among the Chinese nations in the past few decades do not appear to have offset the adverse impact of childhood adversity on multimorbidity.^20^ The food deprivation or food insecurity experienced also reflects the extreme lower end of SES, even in this rapidly developing country, and is possibly generalizable to other middle‐income countries. Future longitudinal research in other settings deserves considerable attention to confirm the reasoning and directionality of these results.

#### 4.1.2 Healthy lifestyle behaviors

The modifiable risk and protective lifestyle behavioral factors associated with multimorbidity identified through the present review included smoking, diet, exercise, and alcohol consumption. Smoking is associated with a higher risk of developing multimorbidity, which aligns with the findings of two included studies that have assessed the effect of lifestyle behaviors on multimorbidity.^21^, ^25^ It is known that smoking leads to oxidative stress and can alter cellular proteins, modifying the expression of genes involved in antioxidant defense, inflammation, cell cycle progression, and apoptosis, causing aging and diseases,^37^ thus an important risk factor for multimorbidity. The Jiangsu Nutrition Study included in this review^19^ found the consumption of fruits, vegetables, and grain products was associated with healthier evolution stages of multimorbidity. The positive impact of the consumption of fruits, vegetables, and whole grains has been reported on a range of isolated chronic diseases, particularly hypertension, cardiovascular disease, diabetes, and stroke.^38^ The possible mechanism was linked to the presence of phytochemicals, dietary fibers, and micronutrients that increase the antioxidant capacity of serum and the formation of endothelial prostacyclin that prevents platelet aggregation and reduces vascular tone.^39^ Fruit and vegetable consumption was also associated with improvement in blood pressure, arterial stiffness, and plasma HDL‐C concentration.^19^, ^40^ Whole grains other than rice and wheat, including oat, corn, sorghum, rye, barley, millet, and quinoa, are less refined and potentially contain more dietary fiber that plays a vital role in the beneficial effects on cardiometabolic health. Other dietary indicator found to be an important determinant of multimorbidity in a Malaysian older population was an insufficient daily nutrient intake, specifically iron, further highlighting the importance of healthy dietary intake.^25^ However, the potential mechanistic links involved in the inverse association between daily iron intake and multimorbidity have yet to be fully elucidated, particularly as clinical manifestations of anemia were not investigated in the study, and only iron deficiency was studied.^25^ It is worth mentioning that our findings with the inclusion of China are possibly an important reflection of trends similar to other upper‐middle‐income countries, such as Malaysia. China's transition to an upper‐middle‐income country has been accompanied by significant changes in lifestyle behaviors, such as dietary habits, and these changes may have had a notable impact on the multimorbidity rates. The traditional diet in China that has been relatively healthier, with a focus on vegetables, rice and lower meat consumption, has been changing with increased affluence. The adoption of a more Westernised diet high in processed foods, sugar and unhealthy fats has led to an increase in diet‐related chronic health conditions, including cardiovascular disease, diabetes and obesity. This is alarming considering our included studies in China with a median follow‐up of 11 years found unhealthy lifestyle behaviors increased the hazard ratios from first cardiometabolic disease to cardiometabolic multimorbidity.^21^ Taken altogether, our review provides some evidence for a need to develop strategies for promoting healthy lifestyles to battle multimorbidity in LMICs (markedly upper‐middle‐income countries) especially as those evidence‐based risk factors are amenable to change through available behavioral‐change interventions. Health policy and clinical practice oriented to the implementation and delivery of behavioral change should also be prioritized.

#### 4.1.3 Obesity

Like findings from HICs, studies included in this review consistently reported that a higher obesity indicator, or when participants crossed the obesity threshold, were independently associated with an increased risk of incident multimorbidity. Obesity has long existed as a major crisis facing the global population; hence, the WHO has officialized obesity as an epidemic. Obesity indisputably impacts an array of significant chronic health illnesses. In fact, 3 out of the 10 leading causes of death worldwide, including ischemic heart disease, stroke, and type 2 diabetes,^41^ were linked to obesity and cardiometabolic diseases. Obesity itself is a chronic and complex condition characterized by excessive fat accumulation in the body, which alters anatomy and physiology, thus resulting in a range of unfavorable metabolic, biomechanical, and psychosocial health consequences.^42^ In parallel to this, it is worthy of further investigation of this strong but modifiable risk for the prevention of multimorbidity. Given the role of childhood adversity in multimorbidity found in our study population, it might be worth exploring in future longitudinal studies if obesity later in life is linked with early‐life undernutrition. The findings also highlighted the importance of weight management for improving health outcomes.

#### 4.1.4 Disability

The potential mechanisms between disability and multimorbidity may be bidirectional, multifaceted, and complex.^22^ On the one hand, individuals with a disability may be facing difficulties in accessing medical care, exacerbating physical illnesses and pains. They may also experience a greater extent of psychological stress, an inability to engage in physical and social activities, and consequently contributing to multimorbidity. In this regard, the mental health consequences of impaired functioning may be a relevant mediator for multimorbidity. On the other hand, a disability could easily be secondary to an existing or undetected health condition. Multimorbidity might further increase disability risk through pathological damage to multiple body systems and organs, resulting in impairment and functional limitations and ultimately disability. These notions provide justification for the consideration of disability as a reliable risk factor for multimorbidity, as well as vice versa.

#### 4.1.5 Dyslipidemia

Evidence from Blümel and coauthors showed that a low HDL‐C at baseline was associated with a higher multimorbidity risk after 30 years.^18^ Dyslipidemia has been reported as a strong risk factor for cardiometabolic diseases,^43^ leading to higher odds of multimorbidity. It might be interesting to know if baseline obesity strongly associated with atherogenic dyslipidemia phenotype is associated with multimorbidity at 30‐year follow‐up. This warrants further exploration in other cohort studies with longitudinal investigations.

### 4.2 Strengths and limitations of this review

There are several strengths of this systematic review. Firstly, our focus on longitudinal studies in LMICs provides the first comprehensive summary of the available long‐term evidence of determinants of multimorbidity in these settings. This review has identified the scarcity of longitudinal evidence available in LMICs and geographical bias in the distribution of multimorbidity research. These may be due to underinvestment in multimorbidity care and research in LMICs, coupled with the challenges of collecting or accessing relevant data. Future research investment is required in other LMICs and other regions. Secondly, this review employed a vigorous and robust approach to search and screen articles for inclusion and review of the selected studies, which should limit selection and extraction bias. Our review that follows a structured approach to gather, evaluate, and synthesise the existing studies not only provides a thorough overview of the available evidence from multiple dimensions of multimorbidity, but also helps to identify the trends, knowledge void, and specific areas where more research is needed. We determined the long‐term risk and protective factors associated with multimorbidity in tandem with their potential underlying mechanisms in LMICs. We also discovered that evidence for MHCs, infectious diseases, undernutrition, and longitudinal studies beyond Asia are in deficiency. Thirdly, this rigorous selection is critical to provide a full opportunity to check the true direction of causality. Longitudinal studies are valuable in their capability to track the study populations over an extended period; hence, our review provides data‐driven insights into how multimorbidity develops and progresses. The results and implications of findings from the review offer a reference point for future multimorbidity research and a starting point for policy formulation for multimorbidity‐related care in LMICs. In this sense, understanding the determinants of multimorbidity is helpful to inform practical strategies to prevent and manage multiple chronic conditions effectively, allocate resources more efficiently, and improve the overall health of vulnerable populations and global health security. Importantly, our findings can also provide insights that are beneficial for HICs dealing with similar issues or looking to support LMICs in addressing their healthcare challenges.

This review has some limitations that warrant discussion. Firstly, notwithstanding efforts made to ensure eligible studies at the global level were included, only a few longitudinal studies in this domain were identified, and nearly all were concentrated in Asia. There was only one study from South America and none from Africa. Longitudinal research into epidemiological aspects of multimorbidity is warranted to build up scientific evidence in regions other than Asia to provide a detailed picture of disease development, with vital implications for community, clinical, and interventions in LMICs. Secondly, although MHCs are NCDs and there is high comorbidity with all chronic non‐MHCs, MHCs were often neglected, as was found in our review. It is imperative that multimorbidity research and management and policies target not only physical health conditions but are also inclusive of MHCs. Additionally, the lack of consensus about multimorbidity definitions and heterogeneity in study designs, exposures, outcomes, and statistical methods undermined the potential to synthesize findings. This calls for greater methodological standardisation while investigating the epidemiology of multimorbidity studies to help improve the precision of estimates, such as being more amenable to meta‐analysis.

## 5 CONCLUSION

This systematic review identified a small but developing body of literature investigating multimorbidity in LMICs longitudinally. Multimorbidity notably remains an unexplored area of research in LMICs, particularly longitudinally and in geographic regions beyond Asia, which warrant future studies. Understanding longitudinal multimorbidity research across different populations in other LMICs will be crucial in navigating the consequent population health, healthcare systems, and economic implications. Cross‐national comparisons across the globe using data harmonization may be useful to provide a comparable view of multimorbidity from different countries. We found a lack of consideration of MHCs, communicable diseases, and undernutrition in the included studies. This calls for a more comprehensive definition of multimorbidity globally. Determinants identified in this review spanned from socio‐economics and social inequities and childhood adversity, to lifestyle behaviors, obesity, dyslipidemia, and disability. To meet the challenges and complexities of providing healthcare to individuals with multimorbidity in LMICs, an integrated‐care and person‐centered approach that accounts for the determinants of multimorbidity is needed. There is also a need for research investment in collecting robust and population‐representative data focusing on multimorbidity risk using a standard classification of multimorbidity and harmonized methodology.

## AUTHOR CONTRIBUTIONS

MMCT, GMT, and MP conceived the idea, designed the study, and supervised the research process. MMCT performed the systematic search and drafted the systematic review protocol and manuscript. MMCT, GMT, MGB, PJMRP, EA, AÁMK, and MP participated in the screening, data extraction, risk of bias assessment, and analysis. GMT provided statistical guidance. MP, GMT, CH, BB, AD, TTS, DM, MGB, and CF critically reviewed the manuscript for meaningful intellectual insight. MP is responsible for the overall content as the guarantor. All authors read and approved the final manuscript for submission. The corresponding authors attest that all listed authors meet authorship criteria and that no others meeting the criteria have been omitted.

## CONFLICT OF INTEREST STATEMENT

MGB, PJMRP, EA, and AÁMK report no relevant disclosures. MMCT was a recipient of the Australian Government Research Training Program Scholarships (Australian Postgraduate Award as well as International Postgraduate Research Scholarship) and The University of Sydney Charles Perkins Centre Early‐ and Mid‐Career Researcher (EMCR) SEED Grant Award. Additionally, MMCT has been awarded three Postgraduate Research Support Schemes by The University of Sydney, New South Wales, Australia. CH is supported by funding from the National Institute for Health & Care Research (NIHR) Global Health Research Group in Homelessness and Severe Mental Illness in Africa (HOPE), and Wellcome Trust funded SCOPE project (Studying the Contexts of Psychosis in Ethiopia to improve outcomes) as the Principal Investigator. CH is additionally supported by the Wellcome Trust‐funded PROMISE project (Psychosis in Malawi) and another NIHR fund for the SPARK project (i.e., cluster Randomized Controlled Trial [RCT] of WHO caregiver skills training for children with developmental disabilities in Ethiopia and Kenya) (Grant/Award number NIHR200842). CH also receives support from the NIHR Global Health Research Unit on Health System Strengthening in Africa (ASSET) via King's College London grant (Grant/Award number GHRU 16/136/54). BB is supported by five NIHR grants. The views expressed in this publication are those of the authors and not necessarily those of the NIHR or the Department of Health and Social Care or Public Health England. AD reported receiving the MRC/NIHR Multimorbidity Prime Pumping Award (Grant/Award number MR/SO28188/1). TTS was supported by the MRC Global Challenges Research Fund (Grant/Award number MR/S014349/1) and the Ministry of Higher Education Malaysia/UK–My Joint Partnership on NCDs grant (Grant/Award number 2019/MR/T018984/1). Malaysian Industry‐Government Group for High Technology (MIGHT) supported both TTS and DM via a Newton Fund Impact Scheme (NFIS)—Newton‐Ungku Omar Fund (Grant/Award number 537084059). DM is additionally supported by the Australian Research Council (ARC) Discovery Project (Grant/Award number DP200102224) and NIHR Global Health Group on Dementia Prevention and Enhanced Care (DePEC) (Grant/Award number 16/137/62) grants. CPF is supported by the *Fundação de Apoio a Pesquisa do Estado de São Paulo* (FAPESP) (Brazilian government agency), MRC (Grant/Award number MR/S004009/1), COSAPI – Brazilian Ministry of Health (Grant/Award number TED176/2020), and *Conselho Nacional de Desenvolvimento Científico e Tecnológico*, CNPq Brasil‐productive research fellowship (Grant/Award number 315593/2021‐0) grants. GMT is funded by two National Institute on Ageing (NIA)/National Institutes of Health (NIH) grants (Grant/Award numbers 1R01AG067621‐01 and 1R01AG067622‐01). MP further received supports from the Michael J Fox Foundation grant and UKRI‐National Health and Medical Research Council (NHMRC) collaboration grant.

## ROLE OF THE FUNDER/SPONSOR

These funding bodies had no role in the design of this review; nor the collection, management, analysis, and interpretation of the data; preparation, review, and approval of the manuscript; nor the decision to submit the manuscript for publication.

## Supporting information


**Table S1.** PRISMA 2020 Checklist.
**Table S2.** Search strategy.
